# Supplementary material for: Model reference adaptive system and pseudo-sliding mode control with exponential reaching law for sensorless-speed control of PMSM
Source: PLoS One. 2025 May 19;20(5):e0321985. doi: 10.1371/journal.pone.0321985 (PMC12088525; doi:10.1371/journal.pone.0321985)
Supplement: Attachment — Submitted filename: Response to Reviewers.docx [file pone.0321985.s002.docx]

**Response report, PONE-D-24-44412**

**Model reference adaptive system and Pseudo-Sliding Mode Control with Exponential Reaching Law for Sensorless-Speed control of PMSM**

The authors would like to express their gratitude to the associate editor and to the reviewers for providing valuable comments to improve the quality of the paper. All comments raised by the reviewers have been addressed in the revised version of the paper. In the new version of the paper, where applicable, corrections, modifications and additions are highlighted in bright green color.

The authors would like to thank the editor for his helpful feedback and suggestions that will help us to make our paper better in a big way. We've carefully thought about your ideas and made the changes that were needed.

**Comments to the author**

**Reviewer #1:**

**1)**	The paper's focus is primarily on the medium to high-speed operation of PMSMs, leaving out important challenges related to sensorless control at low speeds, where accurate estimation of rotor position is difficult. A more balanced discussion on how the proposed system performs across all speed ranges, including low-speed operation, would provide a more comprehensive analysis.

**Author response:**

We acknowledge the reviewer's insightful comment regarding the importance of addressing sensorless control challenges at low speeds for PMSMs. While the primary focus of this work has been on medium to high-speed operations to optimize system performance under these conditions, we recognize that sensorless control at low speeds remains a critical area of development due to the inherent challenges in accurately estimating rotor position at such speeds. The proposed system, primarily designed and validated for medium to high-speed scenarios, relies on certain control techniques that may have reduced estimation accuracy and observability in the low-speed range. To provide a more comprehensive analysis, future extensions of this research will explore adaptations of advanced observer-based methods, such as high-frequency signal injection or improvements in back-EMF observer design, to enhance rotor position estimation and control performance across the entire speed spectrum, including low-speed operation.

In the introduction

According to these studies, employing PMSMs in low-speed range poses some challenges, notwithstanding its intrinsic benefits. A primary difficulty is cogging torque, resulting from the contact between the rotor magnets and the stator slots. This may result in irregular motion at low velocities, causing vibrations and diminished efficacy in precise applications. Thermal management is an additional problem. Operating at low speeds diminishes the efficacy of cooling systems since several processes depend on elevated rates for sufficient airflow. This may result in overheating, especially in high-torque situations. Moreover, current harmonics resulting from suboptimal inverter switching or control errors may impair motor performance and efficiency at reduced velocities.

**2)**	Although the manuscript compares the proposed method with classical ERL-SMC, the range of alternative control strategies is narrow. Control strategies such as Field-Oriented Control (FOC) or other advanced adaptive or predictive controls are not fully explored. The authors should include a broader comparison with state-of-the-art sensorless control methods beyond sliding mode control. This could provide a clearer context for the superiority or trade-offs of the proposed technique.

**Author response:**

We appreciate the reviewer's observation regarding the comparative analysis in our study. While the proposed method has been benchmarked against classical Extended Reaching Law Sliding Mode Control (ERL-SMC) to illustrate its improvements, we recognize that expanding the range of comparisons to include widely used control strategies such as Field-Oriented Control (FOC), adaptive control methods, and model predictive control (MPC) would offer a broader perspective on the efficacy and trade-offs of our proposed approach. FOC, in particular, remains a cornerstone in PMSM control due to its high dynamic performance and stability at varying speeds. Similarly, advanced adaptive controls and predictive controls have shown notable improvements in real-time adaptation and prediction capabilities in sensorless environments. In future work, we aim to conduct a more extensive comparative study involving these state-of-the-art methods to better contextualize the performance, robustness, and limitations of our technique relative to established and emerging sensorless control strategies."

In the results section, the FOC is included to enhance the comparison, and the results are shown in Fig. 20.

**3)**	The MATLAB/Simulink environment used for simulations may oversimplify the complexities of PMSM control, such as measurement noise, real-time computational delays, or implementation constraints. The authors should simulate real-world imperfections such as sensor noise or communication delays to show how the controller handles such challenges. Alternatively, a discussion on the feasibility of real-time implementation and how the system could perform under these conditions is crucial.

**Author response:**

We appreciate the reviewer's observation regarding the limitations of the MATLAB/Simulink environment in capturing real-world complexities, such as measurement noise, real-time computational delays, and implementation constraints. While our simulations primarily focused on demonstrating the core functionality and effectiveness of the proposed control strategy under ideal conditions, we acknowledge the importance of evaluating its robustness against such imperfections.

To address this, we have added a future perspective to the conclusion of our paper, highlighting the necessity for extending this work by incorporating real-time simulations and experiments. Specifically, we propose future efforts to examine the impact of measurement noise, sensor inaccuracies, communication delays, and computational limits using hardware-in-the-loop (HIL) setups or real-time digital simulation platforms. This approach will provide a more comprehensive understanding of the practical viability and resilience of the proposed controller, bridging the gap between simulation and real-world implementation. We believe this stepwise progression will significantly enhance the robustness and applicability of our findings.

**4)**	The paper provides limited detail on how the parameters (e.g., gains of the PI regulator in MRAS or sliding surface design in SMC) are chosen and tuned. The tuning process is often critical to achieving desired performance but is not discussed in depth. The paper would benefit from a more detailed explanation of the parameter tuning process and its impact on system performance. Additionally, the authors should comment on the computational complexity of implementing the proposed system, especially regarding real-time applications.

**Author response:**

We appreciate the reviewer's insight regarding the need for a more detailed discussion on the parameter tuning process and computational complexity. In our work, the parameters for the gains of the PI regulator within the MRAS framework and the sliding surface design in SMC were determined through manual, or hand-tuning, approaches. This method was deliberately selected to maintain system simplicity and avoid the high computational demands and data requirements often associated with optimization-based tuning methods. The hand-tuning process, although less systematic, provided flexibility for iterative adjustments based on observed performance metrics during simulations.

The tuning process primarily focused on achieving a balance between stability, convergence speed, and robustness to disturbances across the operational range. Empirical testing was employed to ensure that gains were neither too aggressive (leading to potential instability or chattering) nor too conservative (resulting in slower system responses). This pragmatic approach aligns with the goal of minimizing system cost and computational complexity, especially for real-time applications.

While we acknowledge that optimization-based techniques may offer a more systematic parameterization, their complexity and resource requirements may not be suitable for cost-sensitive or computationally constrained environments. However, we recognize that exploring hybrid approaches combining manual tuning with automated fine-tuning techniques could enhance the robustness and adaptability of our system. This possibility is noted as a potential direction for future work to strike an optimal balance between performance and computational demands.

**5)**	The manuscript does not address the scalability of the proposed control strategy for larger or more complex systems, such as multi-phase motors or systems with multiple interacting machines. A discussion on the potential challenges and solutions for scaling the proposed control system to more complex or multi-machine systems would enrich the paper's contribution.

**Author response:**

Thank you for raising the important issue of scalability. While our current work focuses on three-phase PMSMs, future research will explore extending our control strategy to more complex systems, such as multi-phase motors and multi-machine configurations. This effort will address key challenges, including increased computational demands, robust decoupling, and inter-machine interactions. By tackling these issues, we aim to demonstrate the adaptability and robustness of the proposed strategy across a broader range of applications. This perspective will form a key direction for enhancing system scalability and performance in diverse, complex setups.

*******************************

**Reviewer #2:**

This manuscript presents the importance of sensorless speed motor drives, particularly in the context of PMSMs, and introduces a hybrid control technique aimed at improving system reliability and performance. The integration of the MRAS and pseudo-sliding mode control, combined with the ARL, presents a novel approach to addressing common control challenges, including uncertainties, dynamic conditions, and chattering issues. However, the writing needs extensively improvements. After carefully reviewing this work, some comments are given below:

**A.Sec.1**

**1. Sec. 1** gives a good breif for the sensorless PMSM systems. However, many references are too out-of-date.

For example, the related works about SMC [31-37] were published between 1983-2023. Only [36] and [37] were published within 5 years. Please consider the following recently published works about SMC techniques.

Yun Zhang, et al. "Vector control of permanent magnet synchronous motor drive system based on new sliding mode control," in IEICE Electronics Express, vol. 20, no. 23, pp. 20230263-20230263, 2023.

Lei Zhang, et al. "PMSM non-singular fast terminal sliding mode control with disturbance compensation," in Information Sciences, vol. 642, pp. 119040, 2023.

Hao Yang et al. "Application of new sliding mode control in vector control of PMSM," in IEICE Electronics Express, vol. 19, no. 13, pp. 20220156-20220156, 2022.

Hao Yang et al. "Generalized super-twisting sliding mode control of permanent magnet synchronous motor based on sinusoidal saturation function," in IEICE Electronics Express, vol. 19, no. 9, pp. 20220066-20220066, 2022.

**Author response:**

**1.** The references that mentioned from the reviewer have been added

**B. Sec. 3**

**2.** The title of Sec. 3 needs to be modified. Maybe

"Designing of the PMSM’s senserless speed obercation using MRAS" a better title.

**Author response:**

**2.** The title have been modified and replaced by "Designing of the PMSM’s senserless speed obercation using MRAS".

**3.** Fig. 2 needs more explaination. Especially the symbols used in this Figure.

Besides, Fig. 2 has no corss-reference. Please refer to Fig. 2 in your context.

**Author response:**

**3.** Fig.2 has been explained by definition of the symbols that used in the figure

As well as, the corss-reference of the figure has been added

**C. Sec. 4**

**4.** In Eq. (20), the symbol of $\Delta T$ is not defined.

**5.** Fig. 5 is not corss-referenced in your context.

**Author response:**

**4.** The symbol of $\Delta T$ in Eq. (20) has been defined:

“Where ; $\Delta T$ represents the time interval over which the change in the variable $\varepsilon\left( t \right)$ is being measured.”

**5.** The corss-reference of Fig. 5 has been added.

**D. Sec. 5**

**6.** Fig. 15 is not corss-referenced in your context.

**7.** The disturbances simulated in the paper are relatively simplistic, focusing on parameter variation and load torque application. Real-world systems often encounter more complex and unpredictable disturbances, and it is unclear how the proposed method would perform under such scenarios.

**8.** The results are based solely on simulations using MATLAB/Simulink. While simulations are valuable, the absence of real-world experimental data raises concerns about the practical implementation of the proposed method, especially under varying environmental conditions or hardware limitations.

**Author response:**

**6.** The corss-reference of Fig. 15 has been added.

**7.** Thank you for your insightful feedback regarding the disturbances tested in our study. We recognize the importance of evaluating control schemes under real-world conditions, which often involve complex and unpredictable disturbances. However, we wish to clarify the rationale behind our disturbance modeling and demonstrate how our approach remains effective even in practical scenarios.

Rationale Behind the Disturbance Scenarios:

Our chosen disturbances, parameter variations, load torque disturbances at different moments, and reverse rotational speed scenarios are not only common in academic research but are also among the most challenging conditions for sensorless-speed control of PMSMs. These scenarios have been well-documented as critical stress tests for evaluating the robustness and adaptability of control systems.

Parameter Variations: Variations in PMSM parameters, such as inductance, resistance and inertia, are essential to simulate since they frequently occur due to temperature fluctuations and component aging. Addressing these variations tests the adaptability of the MRAS and the robustness of Pseudo-Sliding Mode Control (PSMC).

Load Torque Disturbances: Applying sudden and varying load torques at different instants stresses the ability of the control design to maintain performance under dynamic loading conditions. This is a critical test because it mimics scenarios where the motor faces abrupt mechanical load changes in real applications.

Reverse Rotational Speed: Simulating reverse speed with load torque variation represents a stringent test case, challenging the stability and responsiveness of the control design in scenarios where direction and load rapidly change. It ensures that the proposed method can handle complex operational conditions, as would be seen in electric vehicles or other high-dynamic-performance applications.

Defense of Our Method’s Robustness:

Our method, combining MRAS and PSMC with Exponential Reaching Law, is specifically designed to handle abrupt and significant variations in operating conditions. The simulation results clearly indicate:

Rapid and Stable Adaptation: The proposed control system quickly adapts to parameter changes and load disturbances, minimizing torque ripple and overshoots. This rapid response is crucial for high-performance applications and demonstrates the controller’s robustness.

Robust Performance Under Reversal: The effectiveness under reverse speed and simultaneous load disturbances underscores the scheme's ability to maintain stability and efficient performance, even under extreme conditions.

Addressing Real-World Complexity:

While our study used targeted and controlled disturbances to benchmark our approach, the proposed system is inherently equipped to manage more unpredictable, real-world variations. This is due to:

Robustness of Pseudo-Sliding Mode Control: PSMC inherently provides high disturbance rejection and is less sensitive to model inaccuracies, making it suitable for complex real-world scenarios.

Adaptability of MRAS: The MRAS framework continuously adapts to variations, ensuring that the control system remains effective even if disturbances become more unpredictable.

Future Work Considerations:

We acknowledge that real-world conditions often involve multifaceted disturbances. In future work, we plan to extend our study to incorporate additional complexities, such as:

- Multi-source disturbances, including electrical disturbances from power supply variations.

- Environmental effects, like vibrations and temperature variations, that can impact PMSM performance.

- Interaction with external systems to evaluate the robustness of our control design in a more interconnected environment.

**8.** We appreciate the reviewer’s comment on the importance of real-world experimental validation. While our current work is based on extensive simulations using MATLAB/Simulink, we carefully designed these simulations to emulate realistic and challenging operating conditions for PMSMs. This approach provides a solid foundation for assessing the theoretical robustness and performance of our proposed control method.

Justification for Simulation-Based Evaluation:

Complexity and Precision: Simulations in MATLAB/Simulink offer a controlled environment where various disturbance scenarios such as parameter variations, sudden load changes, and reverse speed conditionscan be precisely implemented. This allows for detailed analysis and validation of the control scheme, which may be difficult to replicate with the same accuracy in initial experimental setups.

Comprehensive Performance Assessment: The results obtained highlight key performance metrics, such as torque stability and speed regulation, under diverse and extreme conditions. These findings establish a strong theoretical understanding of the control system's capabilities.

Plan for Experimental Validation:

We acknowledge the importance of validating our control method on actual hardware to address potential challenges, such as environmental variations and hardware limitations. As part of our ongoing and future work, we plan to:

- Implement the proposed control system on a hardware test bench.
- Conduct experiments to evaluate its performance under real-world conditions and verify the simulation results.
- Investigate any discrepancies between simulated and experimental outcomes to further refine the control approach.

**E. Sec. 6**

**9.** Conclusion should be Sec. 6 not Sec. 5. Please renumber it.

**10.** The study focuses on specific PMSM parameters and operational conditions. There is limited discussion on how the proposed control strategy would generalize across different motor types or in applications with significantly different torque or speed requirements.

**11.** Please dicuss some possible disadvanteges about the proposed method.

**Author response:**

**9.** Conclusion has been renumbered.

**10.** Thank you for the insightful comment. Our study focuses on PMSMs because they are widely used in high-performance applications where precision and robustness are critical. The proposed control strategy is designed to handle dynamic conditions effectively, but we acknowledge that different motor types or varying torque and speed requirements could present new challenges. To address this, we plan to explore:

- Generalization Potential: Testing the control method on other motor types, such as induction motors or multi-phase machines.
- Scalability Analysis: Evaluating performance under varying torque and speed profiles to ensure broad applicability.

This ongoing and future research aims to expand the versatility of our control approach beyond PMSM-specific applications.

**11.** We’ve added in the last of the conclusion the disadvantages about the proposed method and future perspective.
