# Supplementary material for: Model reference adaptive system and pseudo-sliding mode control with exponential reaching law for sensorless-speed control of PMSM
Source: PLoS One. 2025 May 19;20(5):e0321985. doi: 10.1371/journal.pone.0321985 (PMC12088525; doi:10.1371/journal.pone.0321985)
Supplement: Attachment — Submitted filename: Response report (4).docx [file pone.0321985.s003.docx]

**Response report, PONE-D-24-44412**

**Model reference adaptive system and Pseudo-Sliding Mode Control with Exponential Reaching Law for Sensorless-Speed control of PMSM**

**The authors would like to express their gratitude to the editor for giving us another opportunity to amend our comments and reconsider the decision from rejection to resubmission after the appeal letter.**

The authors would like to express their gratitude to the associate editor and to the reviewers for providing valuable comments to improve the quality of the paper. All comments raised by the reviewers have been addressed in the revised version of the paper. In the new version of the paper, where applicable, corrections, modifications and additions are highlighted as:

**Green color for the first review.**

**Yellow color for the second review.**

The authors would like to thank the editor for his helpful feedback and suggestions that will help us to make our paper better in a big way. We've carefully thought about your ideas and made the changes that were needed.

Reviewer #1: All comments have been addressed

Reviewer #2: (No Response)

**Comments to the author**

**Reviewer #1:**

1) The primary focus on medium to high-speed operation omits a thorough analysis of sensorless control at low speeds, a critical domain for many PMSM applications.

JALLOL THIS PART MAY BE THE ANSWER TO THE QUESTION OF THE REVIEWEW

PMSMs encounter several constraints in low-speed applications, although their great efficiency and superior performance in diverse environments. A notable restriction is the diminished back electromotive force (EMF) at low velocities, which complicates precise control and may result in unstable functioning. In addition, this negligible EMF necessitating elevated the currents in the motor to attain the appropriate torque, resulting in augmented power losses and heat production

Low-speed applications of PMSMs often require the implementation of sophisticated control strategies or sensorless methods, which may elevate the system's complexity and expense. Furthermore, the torque ripple in PMSMs is often more significant at reduced speeds, which may result in vibrations and noise inside the system. Moreover, the upfront expense of PMSMs, together with the need for advanced drive electronics, may render them less economically feasible than other motor types for low-speed applications.

We may conclude that the use of PMSMs in low-speed applications necessitates sophisticated and intricate control strategies, which will therefore elevate costs. Elevated current is necessitated, which will augment losses and diminish efficiency.

2) The comparative analysis is narrow, and limited to classical ERL-SMC. Field-Oriented Control (FOC) and advanced adaptive or predictive control strategies, which are widely adopted, are notably absent.

3) The paper lacks a systematic discussion on parameter tuning (e.g., gains of the PI regulator and sliding surface design) and computational complexity for real-time implementation.

Thank you for your helpful thoughts. We recognize that talking about adjusting gains of the PI regulator for using a control system in real-time. These factors are key in designing control systems. This study mainly talks about creating and testing a Model Reference Adaptive System (MRAS) and a Pseudo-Sliding Mode Control (PSMC) using an Exponential Reaching Law to control the speed of Permanent Magnet Synchronous Motors (PMSMs) without needing sensors. Tuning the PI limiter and designing the moving surface are important, but they are not part of this task.

Parameter Tuning: This paper does not focus on discussing how to tune PI regulators or build slide surfaces. However, we used recognized rules and methods for their selection to make sure everything ran smoothly and performed well. These methods are mentioned in Section 1 in the introduction, and we can provide more information if you ask. We chose to keep the talk brief to focus on the new ideas in our MRAS and PSMC methods.

Computational Complexity: When designing the suggested controller, we considered how quickly it could work and whether it could be easily implemented. The MRAS and Exponential Reaching Law were selected because they are both efficient to compute and work well.
We think that focusing on MRAS and PSMC in the paper makes the point clear and strong for readers. If the reader thinks it's necessary, we can include extra material or a more detailed talk in an appendix or in a part about future work to explain these ideas further

4) Some references, particularly on sliding mode control techniques, are outdated. Recent advancements should be incorporated to strengthen the literature review.

The references have been updated, thank you for your enhancing this work. We will try our best to ensure strengthen the literature review.

Reference numbers: 13, 15, 16, 18-23, 31-35, 38, 48 have been added and reviewed

*******************************

**Reviewer #2:**

In this revision, all my major concerns have been well addressed. Only some minor comments are raised in this revision. I would like to suggest accepting this manuscript. The leged in Fig. 20 should be given for the three curves in different colors. The same issues were found in other figure, such as Fig. 15 (b). Please check this concern in the whole manuscript.

Thank for you valuable correction. We have correct all manuscript the figures as you mentioned.
